# Supplementary material for: Multiple spatial reference frames underpin perceptual recalibration to audio-visual discrepancies
Source: PLoS One. 2021 May 17;16(5):e0251827. doi: 10.1371/journal.pone.0251827 (PMC8128243; doi:10.1371/journal.pone.0251827)
Supplement: S1 Methods — (DOCX) [file pone.0251827.s001.docx]

# S1 Methods – Experiment 1: Adjusted power calculations

The sample size for the first experiment was determined by an *a priori* power analysis conducted on an initial pilot experiment (see main text; *Experiment 1 – Methods: Participants*). The power analysis and resulting sample size determination were included in our pre-registered design plan. However, the effect size estimate included in this calculation was based on employing a mixed-effects approach for the first-level analyses (regressing participants’ responses against stimulus azimuth). As detailed in the main text (see *Experiment 1 – Methods: Deviations from pre-registration*), we have since adjusted this procedure to instead employ simple regression models in each individual subject. This was necessary as the per-participant mixed-effects parameters produced by the mixed models are biased towards the mean and hence are suboptimal as inputs for further statistical analyses, particularly for the between-subjects comparisons to be made between the first and second experiments. To quantify how this design change might affect our statistical power, we reanalysed the pilot data using the updated regression method and then recomputed our power calculation. We provide full details of this calculation below.

The power calculation critically depends on the fixation-condition by adaptation-duration interaction within the second-level ANOVA of the Ventriloquism Aftereffect (VAE) magnitude estimates. We estimated an effect size for this interaction from the pilot study data, and entered it into a power analysis using G*Power (v3.1; [1]). We selected the *ANOVA: Repeated measures, within factors* option from the *F tests* family. G*Power does not directly support interactions within repeated measures designs, but they can be emulated by setting the *number of measurements* (denoting the number of repeated-measures factor levels) as $\left( k_{1}-1 \right)\times\left( k_{2}-1 \right)+1$, where $k_{1},k_{2}$ give the number of levels within each factor. For the main experiment’s 3×4 design this gives the *number of measurements* as $\left( 3-1 \right)\times\left( 4-1 \right)+1=7$. We additionally set the *alpha* criterion to 0.05, the desired *power* to 0.8, and the *number of groups* to 1 (as the design is fully within-subjects). Finally, we must choose an appropriate effect size specification from the *Options* menu. G*Power offers four options here: “*as in GPower 3.0”*, “*as in GPower 3.0 with implicit rho*”, “*as in SPSS*”, or “*as in Cohen (1988) – recommended*”. The effects of each of these options are described in [2]. The default “*as in GPower 3.0”* option assumes a calculation of the effect size that *doesn’t* take into account the correlation between repeated-measures factors. The effect sizes provided by many statistical software packages in fact *do* account for this correlation, and hence this option will be inappropriate in many cases (including our own). For our purposes, the “*as in GPower 3.0 with implicit rho”* option is functionally equivalent to the “*as in Cohen”* option and so can also be disregarded. The remaining “*as in SPSS”* and *“as in Cohen”* options are both appropriate for our effect size calculation, and differ in terms of whether the non‑centrality parameter is calculated based on the degrees of freedom (*“as in SPSS”*) or the sample size (*“as in Cohen”*). The “*as in Cohen”* option is more stringent and will produce (potentially quite substantially) larger estimates for the required sample size. This option is also recommended by G*Power, but the *“as in SPSS”* is by no means “incorrect” and is also commonly used. Note that the *“as in SPSS”* option refers to the type of effect size specification, and may be used irrespective of whether effect sizes were actually calculated in SPSS.

Using the original mixed-model approach, we obtained an effect size of Cohen’s *f* = 0.91 for the fixation-condition by adaptation-duration interaction. Entering this into G*Power as described above indicates a required sample size of *N* = 18 using the “*as in Cohen”* option, or *N* = 5 using the “*as in SPSS”* option. There is therefore a substantial discrepancy between the outputs of the two options, with neither being necessarily more “correct” than the other. For our pre-registration we chose the more stringent *“as in Cohen”* option and aimed for a slightly larger sample size of *N* = 20. However, sufficient power likely could have been achieved even with a much smaller sample size more in line with the *“as in SPSS”* option outputs.

Turning now to the updated simple regression approach, we find the interaction effect size is reduced slightly to Cohen’s *f* = 0.72. This is due to a slight increase in the variance of the VAE magnitude estimates, in line with the previous mixed-model approach having yielded biased parameter estimates. If we now repeat our power analysis in G*Power, we obtain a recommended sample size of *N = 28* if using the “*as in Cohen”* option, or *N* = 7 if using the *“as in SPSS”* option. Thus, if we exactly follow our pre‑registered protocol using the “*as in Cohen”* option then it appears our selected sample size of *N* = 20 may leave us underpowered for the updated regression approach. However, if we instead use the *“as in SPSS”* option then our existing sample size appears more than sufficient. Clearly there is some substantial ambiguity in the outputs of G*Power’s power calculation, with a four-fold difference in the sample size estimates between the two options, and neither being necessarily more or less “correct” than the other. To help resolve this ambiguity, we repeated our calculations using two further popular software packages for performing power analyses within repeated measures designs: MorePower (v6.0.4; [3]) and PANGEA (<http://jakewestfall.org/pangea/>; [4]). MorePower and PANGEA indicated required sample sizes of *N* = 6 and *N* = 7 respectively. These estimates are therefore more in line with those produced by G*Power using the *“as in SPSS”* option, while those produced with the *“as in Cohen”* option appear somewhat excessive by comparison. Overall, the weight of evidence indicates our selected sample size will still provide sufficient statistical power.

Ultimately, the interaction effect obtained in the main experiment was not significant. Inspecting the VAE estimates (Fig 2b), it is clear that there is little difference between the eye- and head-consistent conditions over adaptation durations, and hence there is not a significant interaction between the fixation-condition and adaptation-duration factors. This is further supported by the corresponding Bayes factors which indicate substantial support for the null hypothesis, both when including (BF_10_ = 0.06; see main text) and excluding the eye+head‑consistent condition (BF_10_ = 0.08; see *S2 Methods*). It is unlikely that simply acquiring a larger sample size would alter this conclusion. Thus, irrespective of any ambiguity surrounding the *a priori* power analyses, in the main experiment we find clear evidence against an interaction effect, and there is little indication of our experiment being insufficiently powered to make this determination.

To summarise, the power calculation reported in our pre-registered design plan was based on an effect size estimate obtained following a mixed-effects modelling approach. This has since been updated to instead employ a simple regression modelling approach, which in turn slightly reduces this effect size estimate. The power calculation described in our pre‑registration is thus no longer strictly appropriate. Repeating the power calculation as originally described but with the updated effect size estimate now suggests our selected sample size of *N* = 20 might leave us slightly underpowered. However, there is substantial ambiguity around these sample size estimates, and alternative calculations yield much smaller estimates that indicate our selected sample size is still more than sufficient. Ultimately, the critical interaction effect in our experiment was not significant, and instead the Bayes factors indicated substantial support for the null hypothesis. Thus, there is no indication of our study being underpowered in practice.

## References

1. Faul F, Erdfelder E, Lang A-G, Buchner A. G*Power: A flexible statistical power analysis program for the social, behavioral, and biomedical sciences. Behav Res Methods. 2007;39(2):175–91. doi: 10.3758/BF03193146.

2. Lakens D. Calculating and reporting effect sizes to facilitate cumulative science: a practical primer for t-tests and ANOVAs. Front Psychol. 2013;4(NOV):1–12. doi: 10.3389/fpsyg.2013.00863.

3. Campbell JID, Thompson VA. MorePower 6.0 for ANOVA with relational confidence intervals and Bayesian analysis. Behav Res Methods. 2012;44(4):1255–65. doi: 10.3758/s13428-012-0186-0.

4. Westfall J. PANGEA: Power analysis for general anova designs. 2016 Feb.
