# Supplementary material for: Multiple spatial reference frames underpin perceptual recalibration to audio-visual discrepancies
Source: PLoS One. 2021 May 17;16(5):e0251827. doi: 10.1371/journal.pone.0251827 (PMC8128243; doi:10.1371/journal.pone.0251827)
Supplement: S2 Methods — (DOCX) [file pone.0251827.s002.docx]

## S2 Methods – Experiment 1: Second-level analyses without eye+head-coherent condition

In the first experiment, the second-level repeated-measures ANOVA analyses included all three levels of the fixation-condition factor (eye+head, eye-, and head-coherent). However, this means the fixation-condition by adaptation-duration interaction is contaminated by the influence of the eye+head‑coherent condition, whilst our hypothesis more critically depends on just the eye- and head-coherent conditions. To address this, we repeated the ANOVA as planned but with the eye+head‑coherent condition removed. Note that this represents a deviation from our pre‑registered design plan.

We first repeated our analyses for the VAE magnitudes. These yielded largely similar results to the main analysis. Whilst the main effect of fixation‑condition was now not significant (F(1,19) = 0.06, *p* = .814, $\eta_{P}^{2}$ < .01, $\eta_{G}^{2}$ < .01, BF_10_ = 0.17), the main effect of adaptation-duration again approached significance (F(2.46, 46.70) = 2.93, *p* = .053, $\eta_{P}^{2}$ = .13, $\eta_{G}^{2}$ = .05, BF_10_ = 2.83), mediated by a significant linear trend (linear: t(57) = 2.86, *p* = .006, quadratic: t(57) = 0.41, *p* = .683; cubic: t(57) = 0.68, *p* = .505). Importantly, the fixation-condition by adaptation-duration interaction remained non‑significant and provided substantial support for the null hypothesis (F(1.96, 37.32) = 0.24, *p* = .785, $\eta_{P}^{2}$ = .01, $\eta_{G}^{2}$ < .01, BF_10_ = 0.08).

We next repeated our analyses of the spatial gain differences. These yielded substantially similar results to the original analyses: there was no significant main effect of fixation‑condition (F(1,19) = 0.39, *p* = .542, $\eta_{P}^{2}$ = .02, $\eta_{G}^{2}$ < .01, BF_10_ = 0.19), no significant main effect of adaptation-duration (F(2.00, 37.98) = 0.70, *p* = .500, $\eta_{P}^{2}$ = .04, $\eta_{G}^{2}$ = .02, BF_10_ = 0.11), and no significant fixation-condition by adaptation-duration interaction (F(2.66, 50.62) = 0.92, *p* = .429, $\eta_{P}^{2}$ = .05, $\eta_{G}^{2}$ = .01, BF_10_ = 0.15).
