# Supplementary figures and images for: Multiple spatial reference frames underpin perceptual recalibration to audio-visual discrepancies
Source: PLoS One. 2021 May 17;16(5):e0251827. doi: 10.1371/journal.pone.0251827 (PMC8128243; doi:10.1371/journal.pone.0251827)

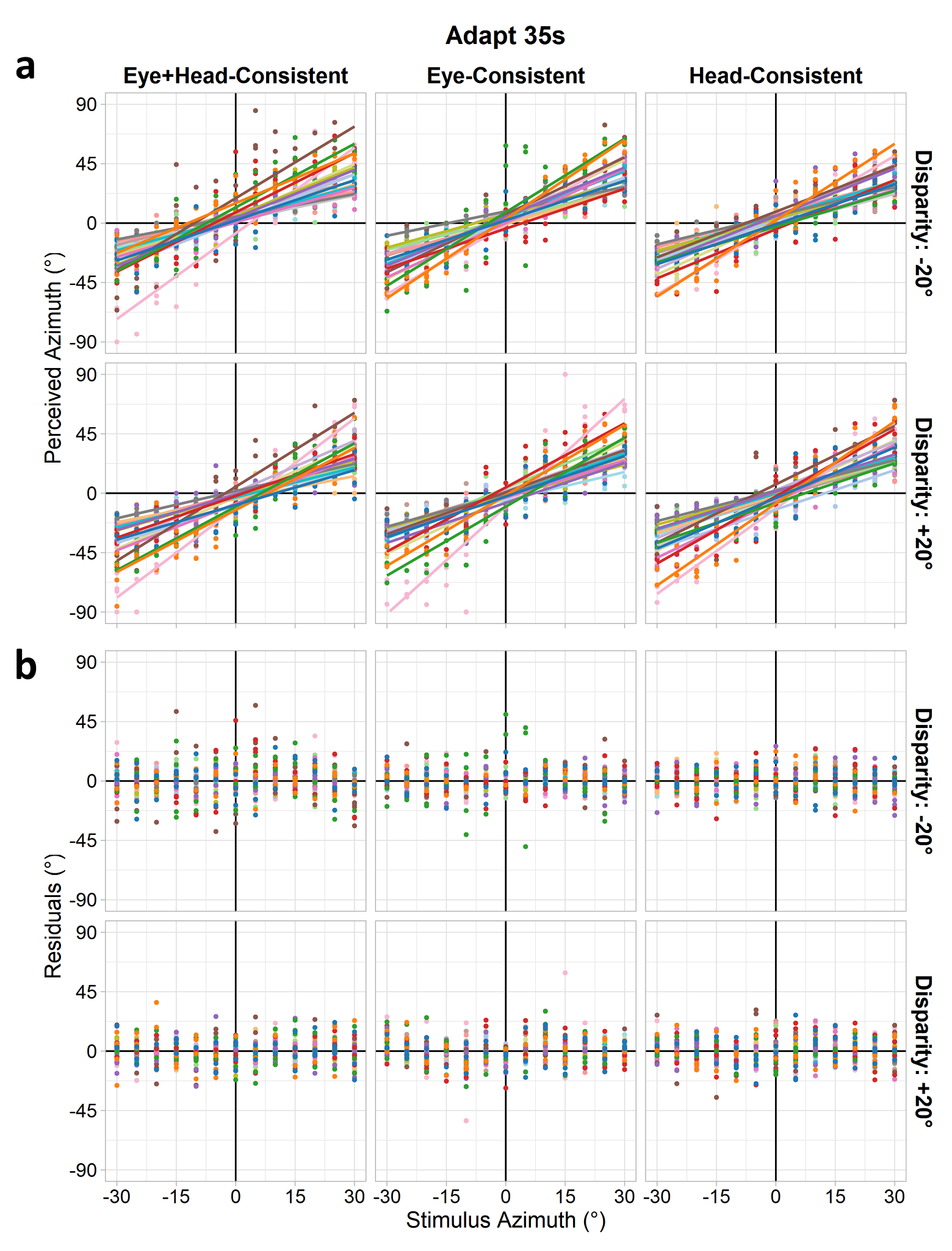

Supplement: S1 Fig — (a) Participants’ perceived stimulus azimuth plotted against actual stimulus azimuth, following 35 seconds of adaptation to audio-visual pairs spatially offset by -20° (leftward audio offset; top row) or +20° (rightward audio offset; bottom row), and for each fixation condition (eye+head-, eye-, and head-consistent; across columns). Data were entered into a series of linear regression analyses for each participant and condition separately. (b) Corresponding model residuals. Data points and model fits are colour-coded by participant. (TIF) [file pone.0251827.s003.tif]

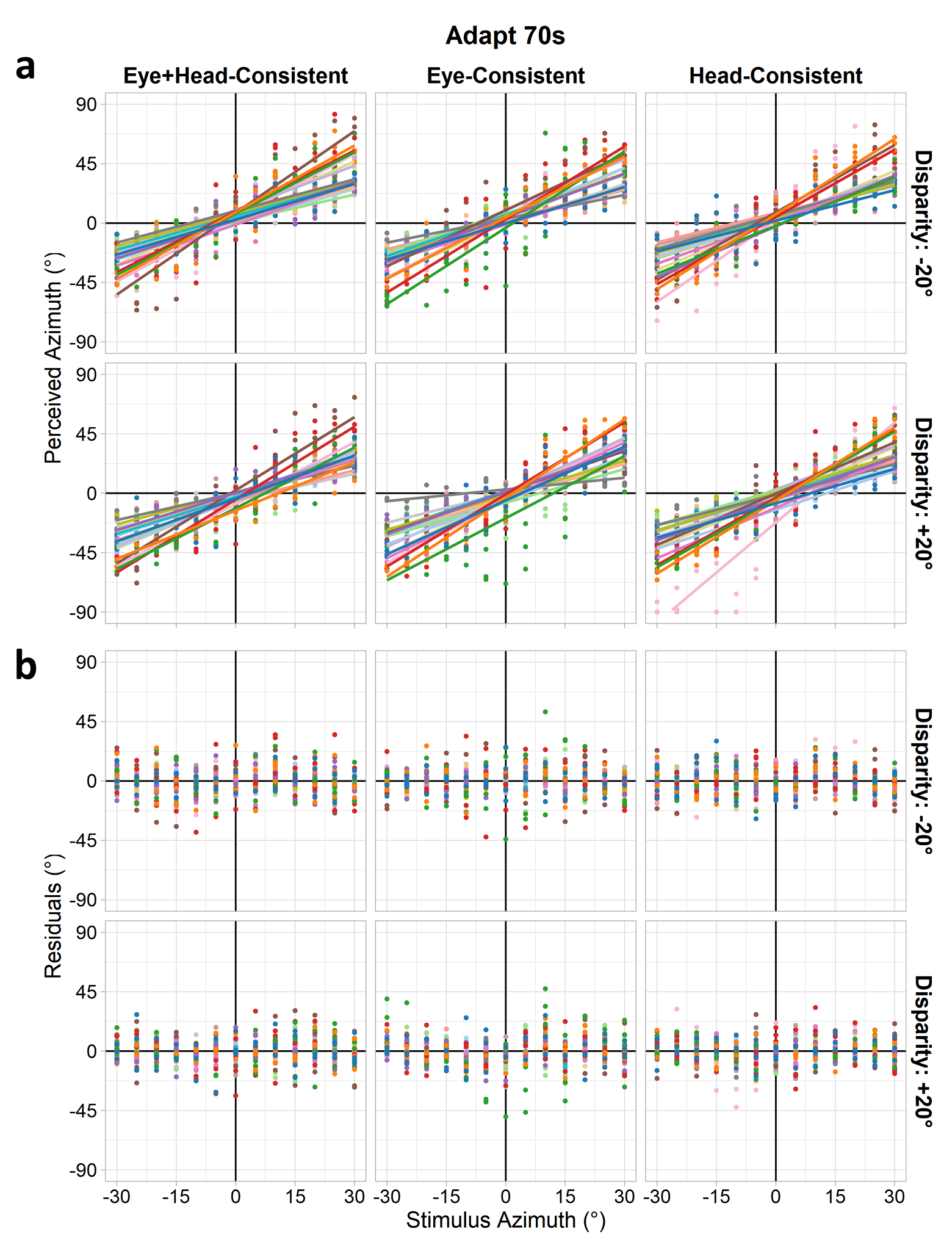

Supplement: S2 Fig — As per S1 Fig, but following 70 seconds of adaptation. (TIF) [file pone.0251827.s004.tif]

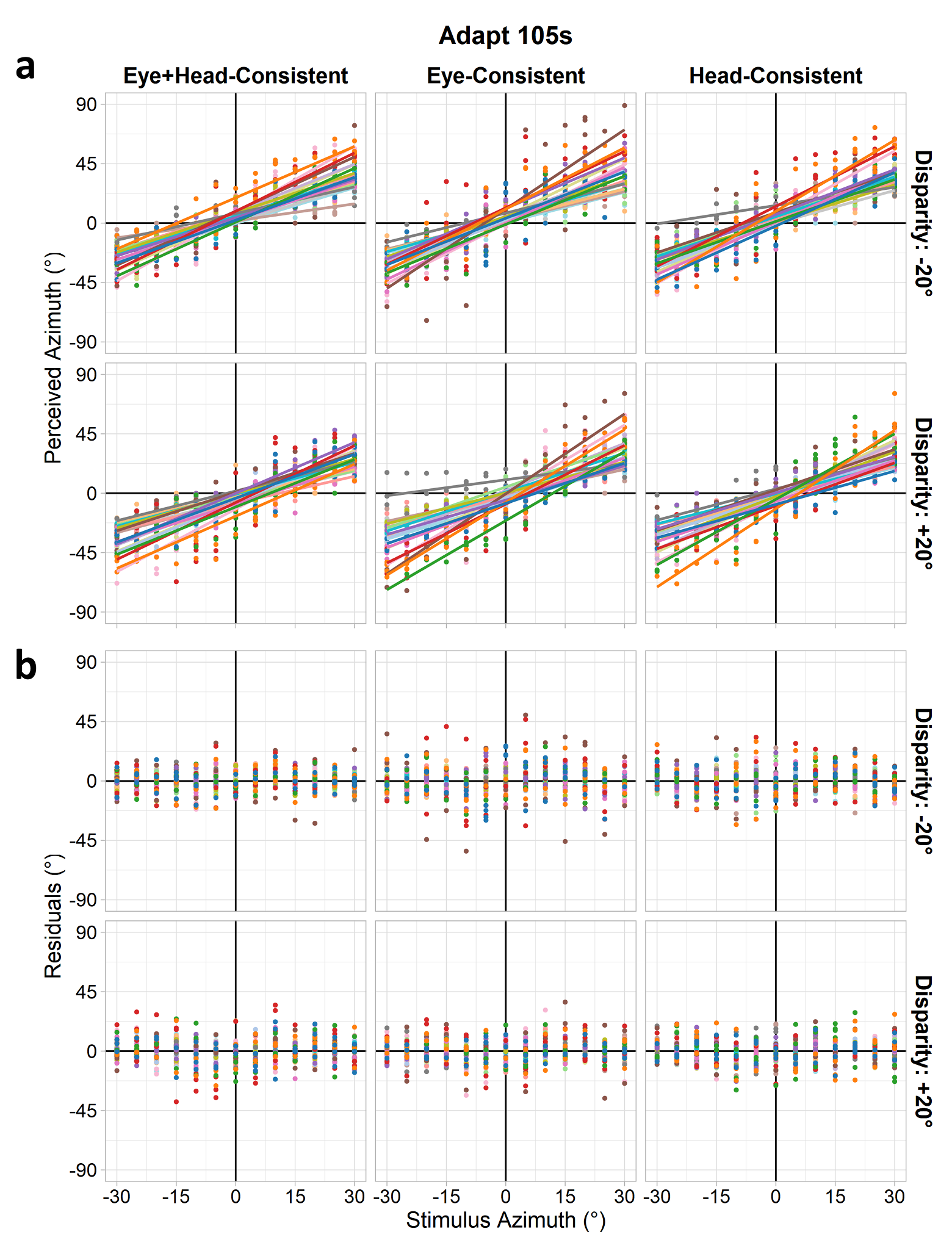

Supplement: S3 Fig — As per S1 Fig, but following 105 seconds of adaptation. (TIF) [file pone.0251827.s005.tif]

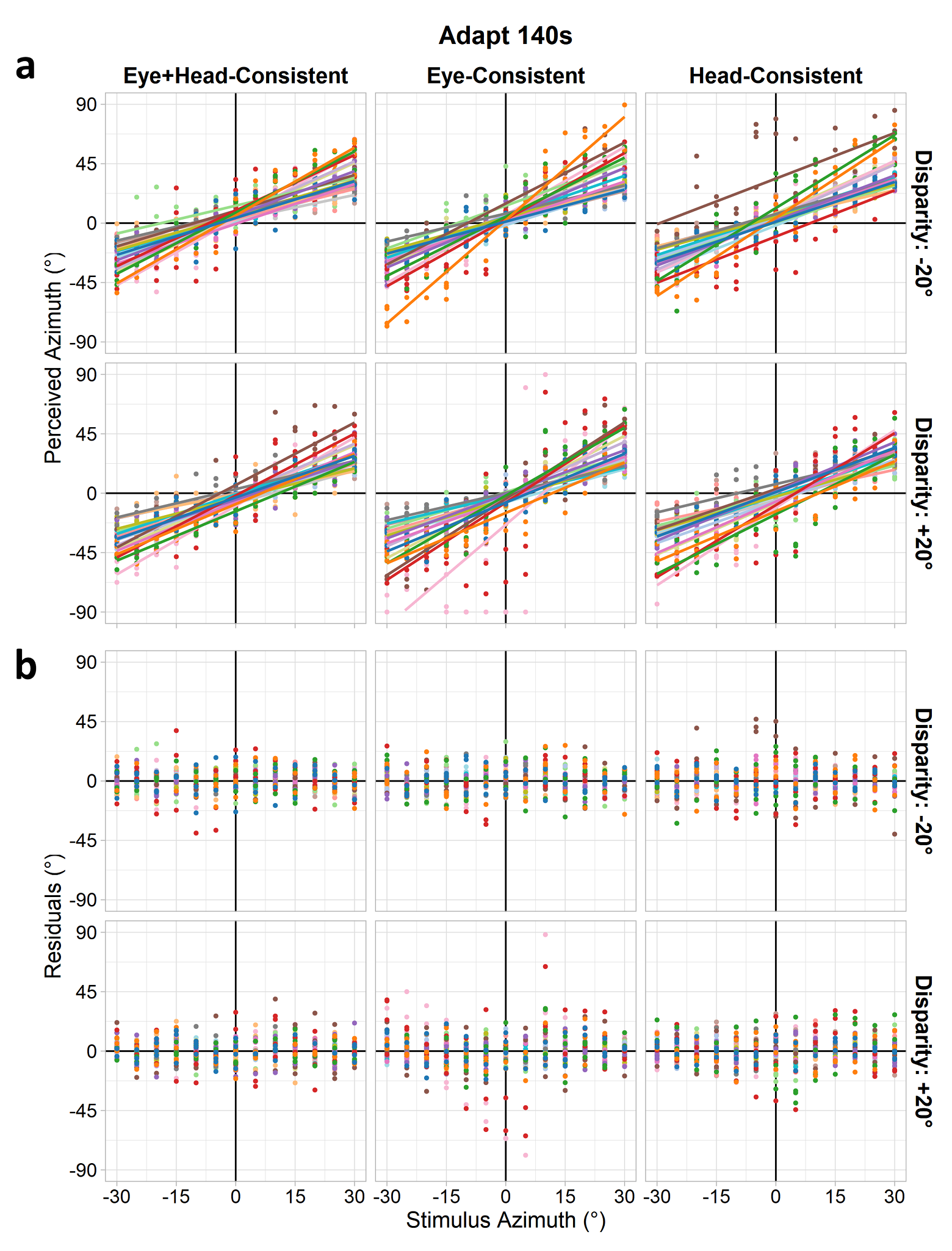

Supplement: S4 Fig — As per S1 Fig, but following 140 seconds of adaptation. (TIF) [file pone.0251827.s006.tif]

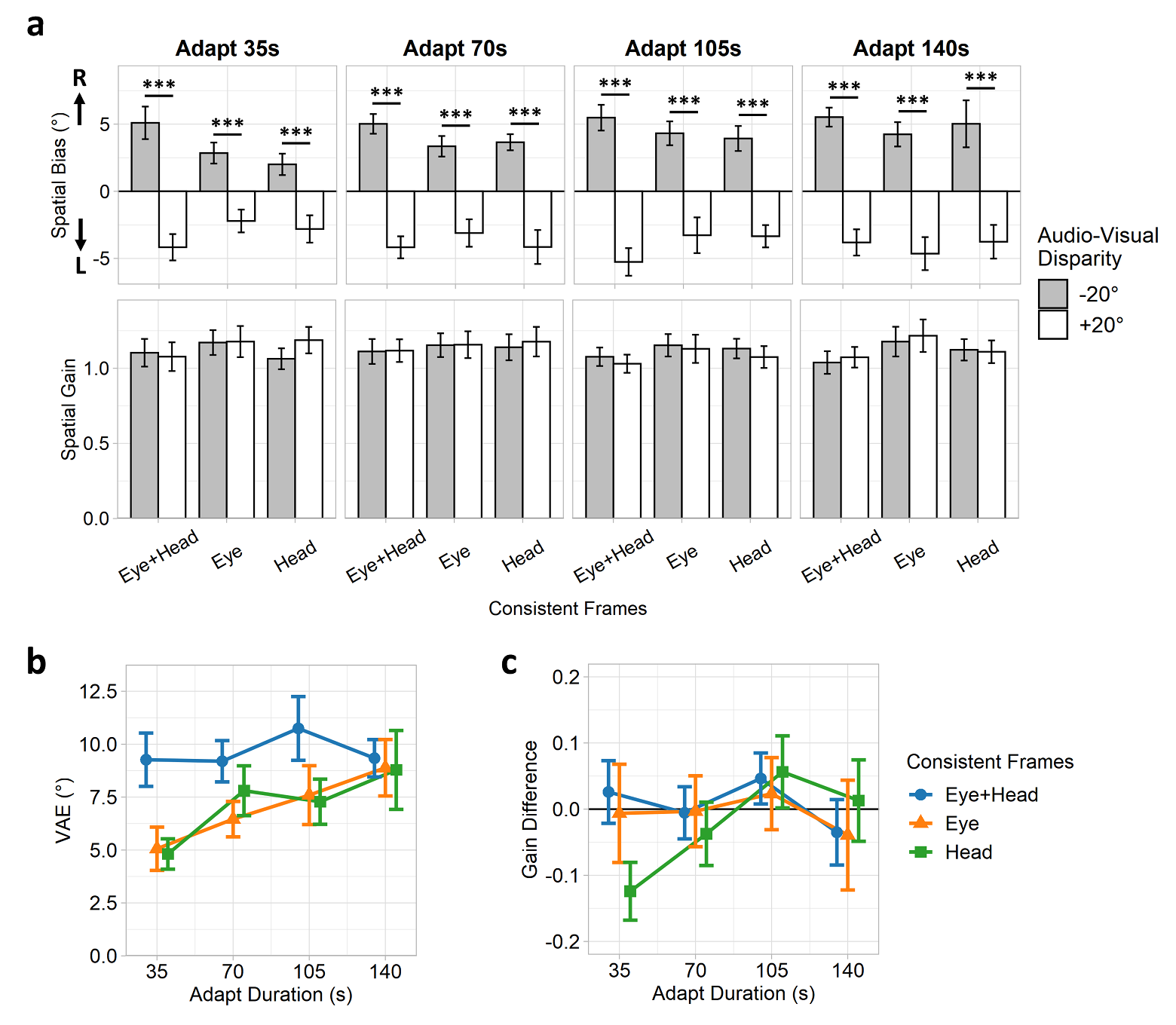

Supplement: S5 Fig — Parameter estimates obtained from mixed-effects regression models allowing random intercepts and slopes over participants. Results appear near-identical to those of the standard regression analyses (cf. Fig 2). (a) Spatial bias (intercept) and gain (slope) coefficients for each condition. Error bars indicate standard errors of the coefficients. (b) VAE magnitudes and (c) gain differences, quantified by contrasting spatial bias and gain coefficients between adaptation disparities (-20° > +20°). Positive VAE magnitudes indicate spatial recalibration in the direction of the visual offset. Error bars indicate standard errors of the mean. (TIF) [file pone.0251827.s007.tif]

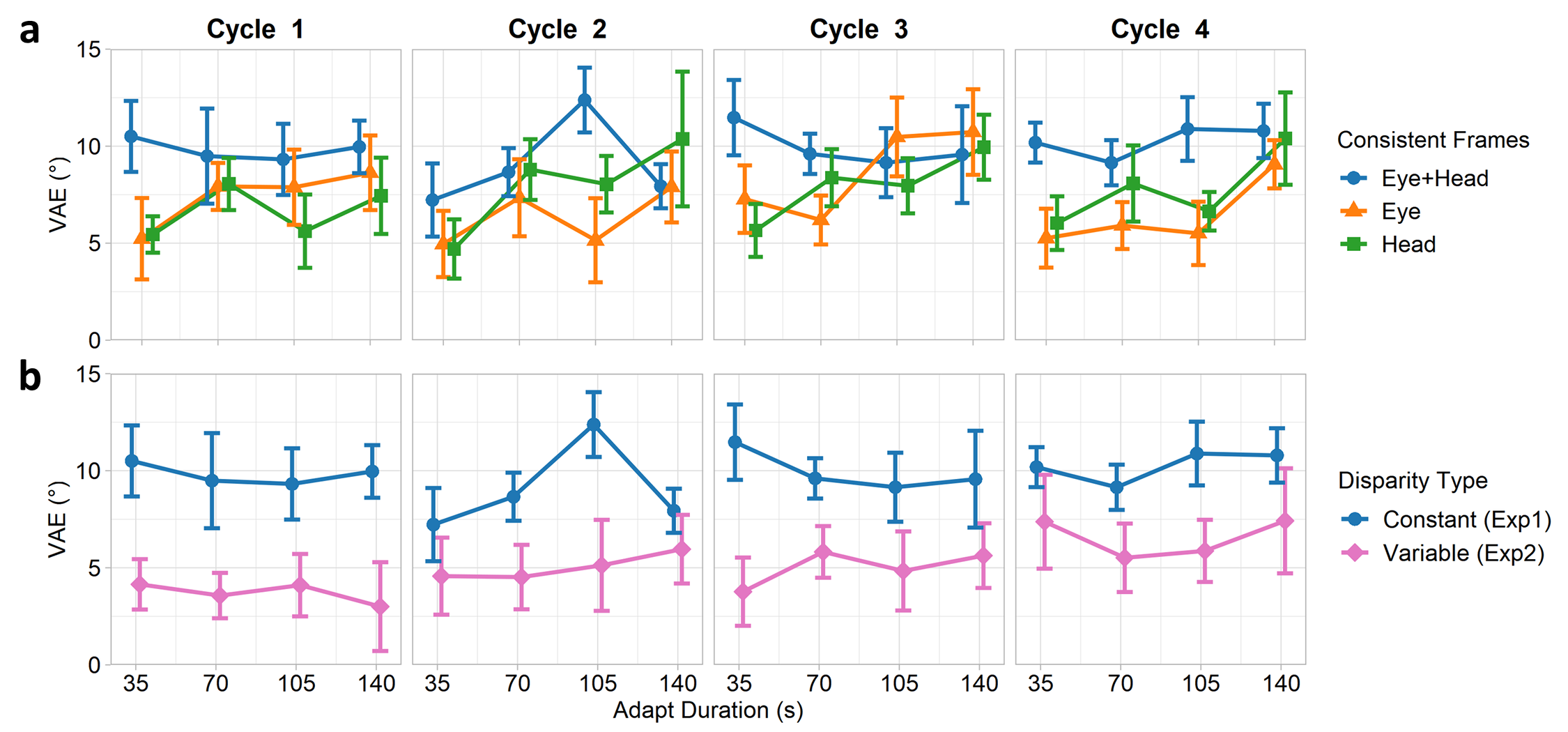

Supplement: S6 Fig — (a) Experiment 1 estimates. A three-way repeated-measures ANOVA revealed a significant main effect of fixation-condition (F(1.62, 30.69) = 5.04, p = .018, BF10 = 1519.74), but no significant main effects of adaptation-duration (F(2.26, 42.93) = 2.11, p = .128, BF10 = 2.11) or cycle-number (F(2.73, 51.81) = 2.10, p = .117, BF10 = 0.01). No interactions were significant (all p >.05, all BF10 < 0.33). (b) Experiment 2 estimates. A three-way mixed-design ANOVA revealed a significant main effect of disparity-type (F(1, 30) = 9.82, p = .004, BF10 = 9.53), but no significant main effects of adaptation-duration (F(2.48, 74.29) = 0.16, p = .896, BF10 = 0.01) or cycle-number (F(2.25, 67.36) = 1.76, p = .175, BF10 = 0.05). No interactions were significant (all p >.05, all BF10 < 0.33). (TIF) [file pone.0251827.s008.tif]

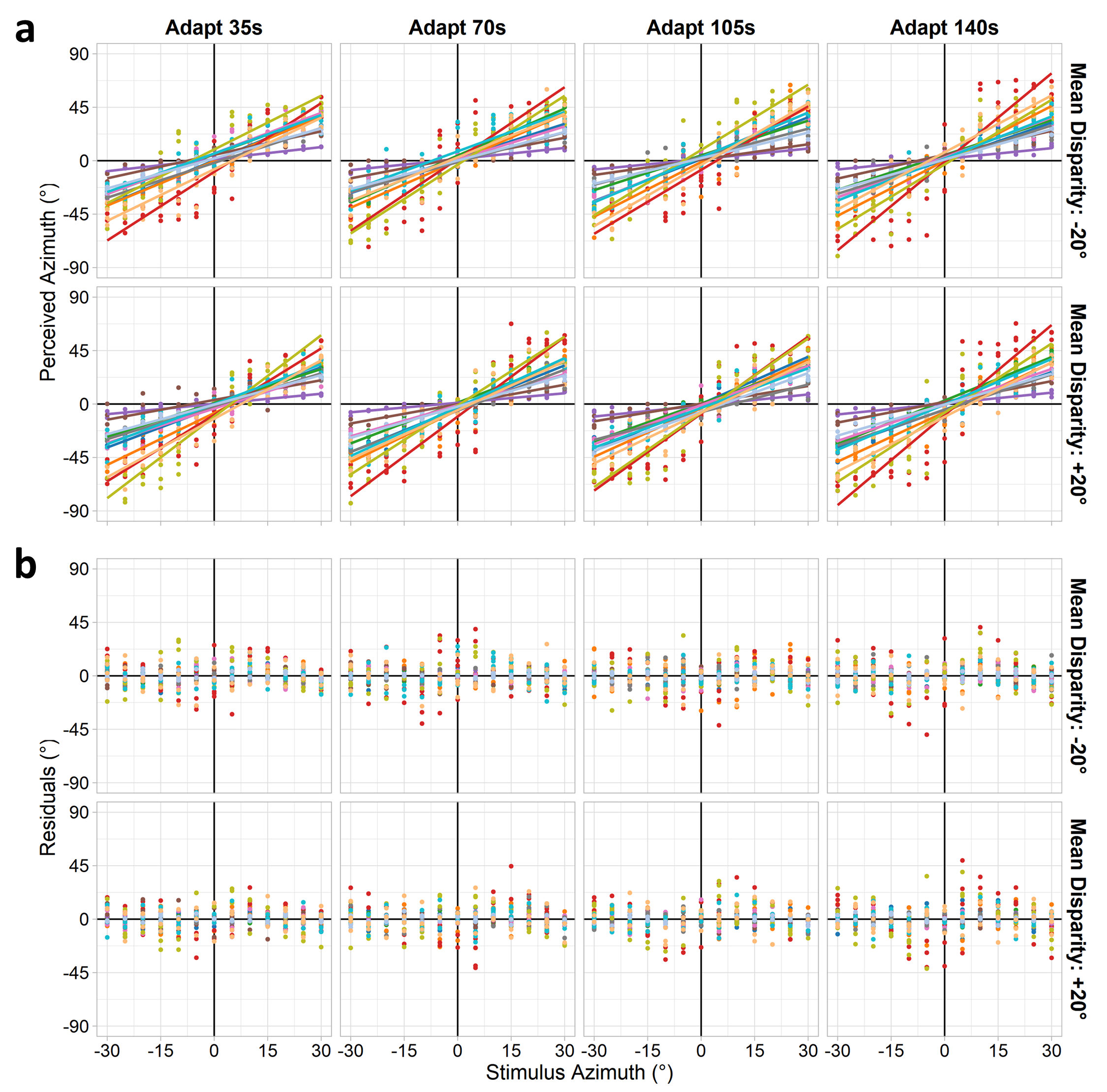

Supplement: S7 Fig — (a) Participants’ perceived stimulus azimuth plotted against actual stimulus azimuth following adaptation to audio-visual pairs spatially offset by an average of -20° (leftward audio offset; top row) or +20° (rightward audio offset; bottom row). Adaptation durations are represented across columns. Data were entered into a series of linear regression analyses for each participant and condition separately. (b) Corresponding model residuals. Data points and model fits are colour-coded by participant. (TIF) [file pone.0251827.s009.tif]

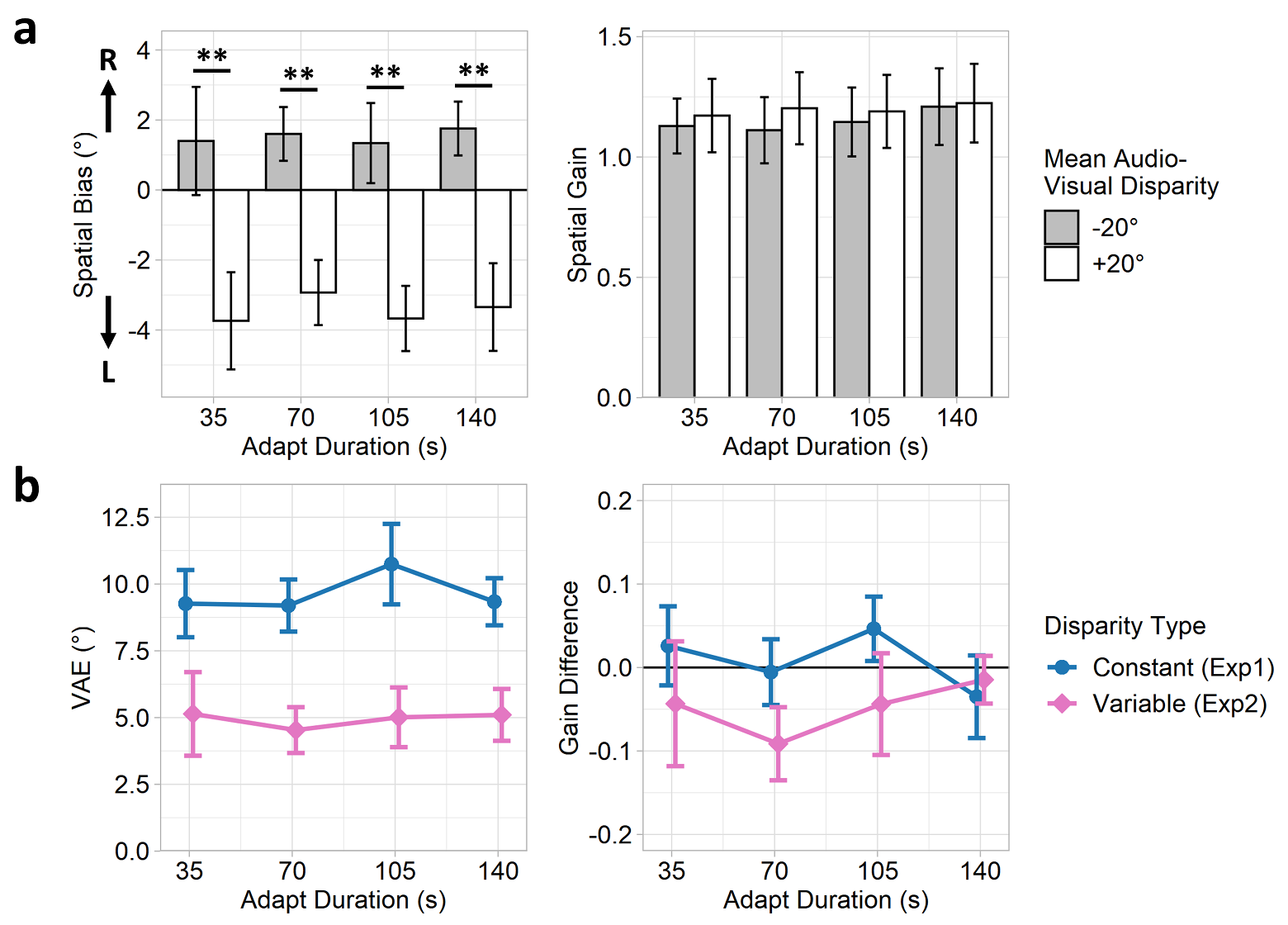

Supplement: S8 Fig — Parameter estimates obtained from mixed-effects regression models allowing random intercepts and slopes over participants. Results appear near-identical to those of the standard regression analyses (cf. Fig 3). (a) Spatial bias (intercept) and gain (slope) coefficients for each condition. Error bars indicate standard errors of the coefficients. (b) VAE magnitudes and gain differences, quantified by contrasting spatial bias and gain coefficients between adaptation disparities (-20° > +20°). Values are shown for both constant (blue; Experiment 1: eye+head-consistent condition) and variable disparities (pink; Experiment 2). Positive VAE magnitudes indicate spatial recalibration in the direction of the mean visual offset. Error bars indicate standard errors of the mean. (TIF) [file pone.0251827.s010.tif]
