# Supplementary material for: Multiple spatial reference frames underpin perceptual recalibration to audio-visual discrepancies
Source: PLoS One. 2021 May 17;16(5):e0251827. doi: 10.1371/journal.pone.0251827 (PMC8128243; doi:10.1371/journal.pone.0251827)
Supplement: Attachment — Submitted filename: PlosOne_WatsonAkeroydRoachWebb_ReviewerResponse.docx [file pone.0251827.s011.docx]

This study describes an experiment in which the authors attempted to determine whether the ventriloquism aftereffect (VAE) operates in eye-centered (EC) or head-centered (HC) reference frames, and over what timescales. The authors claim they developed a new paradigm for selectively manipulating the contribution of EC vs. HC reference frames (RFs). I don’t think the new paradigm is providing the dissociation the authors claim it does. Moreover, it is causing many other differences between the conditions, which might easily explain the observed effects, and which are arguably much more plausible than the RF explanation. Therefore, while the data and statistics are valid (the experimental procedures have some shortcomings, e.g., no eye tracking), the conclusions about the reference frames cannot be supported by these data.

Specifically, the main difference between the new paradigm and the previous studies of RFs of VAE is that here the dissociation between RFs is based on manipulations during the adaptation trials, while the test trials are identical across the conditions (in Kopco et al studies , it was the difference in FP locations used during test trials that provided the dissociation). Here, three conditions are used, with head (and thus HC RF) always fixed. In the E+H coherent condition, eyes are fixed at 0, and the stimuli are presented at different locations with a fixed A-V disparity. In the H-only coherent condition, the stimuli are the same, but the eyes are always following the V-component. In the E-only coherent condition, the V-components change their location, the eyes follow them, but the A-component is always played from the same location (+/-20degs), thus the AV-disparity is actually changing widely from trial to trial.

I think this design has a logical flaw. Specifically, it assumes that the A-components are always represented in HC RF (a fact that the authors never mention), while only the V-components might be either kept in the native EC RF or converted to the auditory HC RF. And that is not logical: for the system to work, the adaptation has to occur IN A COMMON RF, and that can be EC or HC (or mixed). So, either the A-signals get converted to EC RF, or the V-signals get converted to HC RF (or something half-way). Otherwise, for stimuli that actually preserve their physical AV disparity, the system would report that their “Eye-only” disparity is jumping!?!?!, and there would be no way for the system to consistently associate A and V components and know how to adapt the A-representation based on the V signal. This “common RF” requirement is fulfilled by the H-only condition, in which presumably the V-signals are turned into HC RF (ie., the same RF as the A-components), and then the AV-disparity is preserved, and the only difference between the HC and EC representations is that the stimuli are presented from a fixed location in the EC RF while they are presented from varying locations in the HC RF. The E-only condition should work in the authors’ idea because the A-components would be kept in HC RF and the V-components in the EC RF, resulting in EXACTLY NOT SOLVING THE PROBLEM THE SYSTEM NEEDS TO SOLVE – because physically misaligned signals would be perceived as aligned, and vice versa!?!?! In order to fulfil the requirement, the condition would have to be such that the V-component would be always at 0, the A component at +/-20, and the eyes would move by themselves. That would also make this condition the complement of the H-only condition. However, even then, the difference between the conditions wouldn’t be in which RFs they are consistent with, but only in whether the stimuli are at the same location in HC RF and at varying locations in EC RF, or vice versa.

This is an important point. Our experiment does critically assume that reducing the spatial coherence between audio and visual signals will lead to a reduction in the magnitude of the VAE, but we had not explicitly characterised the extent of this reduction. Indeed, a recent study published after our initial submission (Bruns *et al.* 2020, European Journal of Neuroscience) indicates that reliable VAEs may be elicited even when audio-visual disparities are variable over time. Consequently, we have collected additional data to explicitly test the effect of variable versus constant audio-visual disparities on the VAE, within the context of our experimental paradigm.

These data are now included in Experiment 2. We reproduced the conditions of the eye+head-coherent condition of Experiment 1, in which participants maintain central fixation throughout and the auditory stimuli are positioned relative to the visual stimulus location. This ensures that both eye- and head-centred visual reference frames are matched. However, we now vary the magnitude of the audio-visual disparities from trial to trial, such that they are drawn from a distribution with a mean of ±20°, but now also include disparities above and below this. Importantly, this reproduces the distribution of audio-visual disparities that would have been expected using visual signals from the *incoherent* reference spaces in Experiment 1 (head-centred space in the eye-coherent condition, and eye-centred space in the head-coherent condition). We find that reliable VAEs are indeed elicited following adaptation to variable disparities (Experiment 2), but that these are reduced in magnitude compared to those elicited by the constant disparities in the eye+head‑coherent condition of Experiment 1. This indicates that the VAEs observed in Experiment 1 will have been primarily driven by visual signals from the *coherent* reference space, although signals from the incoherent reference space may still have made lesser contributions. Importantly, this reinforces the conclusions of our first experiment: that the VAE is underpinned by visual signals from both eye- and head-centred reference spaces. Furthermore, it is unlikely that the effects observed in the first experiment could have been substantially due to non-spatial factors (such as task differences).

Auditory signals are necessarily acquired in head-centred coordinates, while visual signals are acquired in eye-centred conditions. There is substantial evidence for spatiotopic transformations of visual signals, and it is easy to see how such representations could be combined with head-centred auditory signals. The procedure by which eye-centred visual signals may be combined with the head-centred auditory signals is less obvious. Our results (and those of Kopčo et al, 2009) suggest that eye-centred visual signals might simply be directly mapped onto the head-centred auditory signals (e.g. so that foveal locations are mapped to 0° auditory azimuth regardless of eye position). As the reviewer points out, real-world multi-modal objects would typically be spatially coherent in world-centred rather than eye-centred co-ordinates. Integrating visual signals directly from eye-centred frames then would lead to incoherent variations in audio-visual spatial cues, and so the functional purpose of such a system does appear counterintuitive. Speculatively, such a system could potentially allow correcting audio-visual disparities that arise from eye movements, or alternatively might not be functional at all and simply reflects an inevitability of the system architecture.

Given the importance of these issues, we have made a number of changes to our manuscript to clarify them:

- We have included the results from the second experiment (lines 390-516), and updated the General Discussion to discuss them (lines 562-589).
- We have added 2 extra paragraphs to the discussion. The first (lines 590-605) discusses how head- and eye-centred visual signals may be combined with head-centred auditory signals. The second (lines 606-624) discusses the possibility of eye-centred transformations of auditory signals and why we feel this is unlikely to explain our results.
- We have updated our terminology throughout to refer to the conditions as “eye+head-coherent”, “eye-coherent”, and “head-coherent” to make clearer the distinctions between our conditions.
- We have also updated our terminology throughout to explicitly refer to auditory signals as being head-centred, and visual signals as being variously eye- or head-centred.

The authors find that the induced VAE bias is smaller in the E-only and H-only conditions than in the E+H, and that is supposed to be the evidence that the adaptation RF is mixed. However, in addition to the dissociation not working, as I described above, the result has many more plausible explanations. Specifically, for the E-only condition, since the physicall AV disparity is varying from trial to trial (including trials in which the sign of the disparity switches, i.e., it is in the opposite direction) it is very much expected that the net observed VAE is smaller, simply because the system cannot find an alignment that would be consistent from trial to trial. The explanation of the weakened VAE in the H-only condition would suggest that the EC representation plays a role, because in EC representation the adapted region is shrunk to a single location. But, here, again, are more plausible explanations, having to do with the fact that the subjects actually move their eyes from trial to trial. E.g., is likely that accuracy with which the subjects saccade to the V-component varies from trial to trial, and so the relative location of the A-component in the EC RF varies, thus resulting in noise in the estimated AV-disparity to which the system wants to adapt. Having an eye tracker to show how accurately and reliably the subjects performed the task is critical here. Moreover, Pages & Groh (https://www.ncbi.nlm.nih.gov/pmc/articles/PMC3757015/) showed that the observed effect is critically dependent on when the V signal appears with respect to the A signal. So, that in itself, can be critical here, given that the authors didn’t track the saccades. Finally, Kopco et al. (https://www.ncbi.nlm.nih.gov/pmc/articles/PMC6707804/) showed that adaptation in the auditory representation can occur even with aligned AV stimuli, depending on the FP locations. So, not knowing where the eyes were and not having an AV aligned condition (ie, with disparity of 0), allows even such adaptations to provide the explanation for the observed “reduction in VAE”.

As stated above, we now provide additional data speaking to this issue. If the differences between the eye-coherent/head-coherent and the eye+head‑coherent conditions were simply due to task differences (e.g. in eye movements, or attentional demands), then we would not expect a difference between the VAEs elicited in Experiment 2 and the eye+head‑coherent condition of Experiment 1. Here, the experimental conditions are matched except for the constancy/variability of the audio-visual disparities. The fact that we do find a reduction in VAE magnitudes with variable (relative to constant) disparities suggests that the effects observed in Experiment 1 cannot be fully explained by non-spatial factors.

The audio-visual signals were always presented synchronously together. In addition, a one second interval was included between each set of 5 presentations (i.e. between each azimuth change) to give participants sufficient time to make their saccade before stimulus presentation resumed, such that subjects should always have been fixating the correct location during stimulus presentation. Our paradigm is therefore comparable to the “synchrony-and-feedback” condition of Pages & Groh (2013). We have added points to the Methods to clarify these details (lines 204, 208-210).

We explored the possibility of performing eye tracking, but doing this unobtrusively within our experimental setup would have been impractical. With this issue in mind, we chose discrete azimuths spaced widely apart (10°) for the adaptation phase: although subjects may not have perfectly fixated the required locations, the magnitude of fixation deviations would be relatively small compared to the steps between the locations. Furthermore, as stated above, the results of Experiment 2 now indicate that differences between conditions in Experiment 1 are primarily driven by spatial factors. Thus, although small fixation deviations may still have introduced additional sensory noise, this factor alone cannot account for the results in Experiment 1.

So, while the data are in principle valid (if the lack of eye tracking and of AV aligned condition is ignored), the paper would need to completely change its scope. In particular, for the E-only condition, it would have to be about how varying AV-disparity changes VAE (with the additional nuisance of moving the eyes). For the H-only, it should be about how the range of stimuli in EC vs HC frames affects the strength of the VAE. But, in order to do the same for the E-only condition, and to say at least something about HC vs EC RFs, the E-only condition would need to be modified as I describe above (fix the A and V locations, move eyes).

As stated above, the additional results presented in Experiment 2 indicate non-spatial factors cannot account for the effects observed in Experiment 1. This supports our original conclusions: that the VAE can be driven by visual signals from both eye- and head-centred reference spaces.

Minor comments:
Raw data plots in Figs S1 to S4 are hard to interpret with individual slopes and then individual residuals. Why not plotting a simple across-subject mean responses (or response bias re. A-component location) with SEM so that the reader sees the raw data and can assess how much bias there was in them? The mixed modeling might be hiding extreme amount of noise and variability. Also, it would be easier to see whether the data are linear, or, whether adaptation is local (in particular for the E-only condition), as reported in the Kopco et al. RF VAE studies. Also, why are the supplement Figs called “S1 Fig” etc instead of “Fig S1” etc? I would suggest the latter option.

We have now updated our methodology to apply fixed-effects modelling within each individual subject, rather than a mixed-effects approach across subjects. This was done to address limitations with the mixed-effects approach in which the per-subject parameter estimates are liable to be biased towards the mean, which may have been especially problematic for the between-subjects comparisons between experiments (see Experiment 1 Methods: Deviations from pre-registration). In practice, fixed- and mixed-effects approaches yield near identical estimates (Fig2 vs S5 Fig, Fig3 vs S7 Fig). We still provide scatter plots in S1-S4 Figs (and now also S6 Fig for Experiment 2), but these now show the per-subject fixed-effects fits. To aid visualisation, we have updated these to colour code the fits and data points by the subject ID. Plotting a cross-subject mean response (e.g. averaging within each auditory azimuth) would simplify the visualisation, but would also hide between-subject variability, which we feel would be undesirable. Plotting an average response bias against each auditory azimuth would also be possible, but would not accurately reflect the fixed-effects modelling procedure actually used to analyse the data (in which a global bias parameter across all azimuths is estimated), and so again seems undesirable.

The “S* Fig” convention (as opposed to “Fig S*”) is simply the preferred the style for the PLOS journals.

L187 & 192 To stress that you actually assumed the A-component position is always HC, you should say “the auditory head-centered position”

We have made this change.

L215-217 Sentence “Audio-visual pairs…” When did this happen? At the beginning of each block, or of each adaptation phase? And what does the (7) refer to? Azimuths?

Audio-visual pairs were presented during the adaptation phases. Each block comprised 4 adaptation phases, interleaved with 4 corresponding test phases. We have updated the Methods to clarify the audio-visual stimuli refer to *each* adaptation phase (paragraph starting line 204). The “(7)” is an in-line citation to Recanzone (1998) which employed a similar method of presenting stimuli repeatedly at each location; we have moved this citation to the end of this sentence to avoid any confusion (line 208).

L238-240 I’m not sure I understand how did the adaptation phases differ depending on duration. Were there more “cycles” in the longer adaptation-duration conditions (eg 35 vs 140s)?

Yes, one full pass over all the adaptation azimuths takes 35 seconds. The 35, 70, 105, and 140s adaptation conditions thus comprise 1, 2, 3, and 4 passes over the azimuths respectively. We have updated the Methods to clarify this (lines 219-221).

L245. It would be useful to state what was the average duration of a block, session and the whole experiment.

We have updated the Methods to include the average block durations for each experiment (lines 241-243; lines 433-435). The duration of sessions and the experiment as a whole would vary from subject to subject (e.g. depending how long they take to complete each test phase), and the duration of sessions would also vary according to the randomisation of the adaptation-duration conditions, thus we cannot provide reliable estimates for these.

L330: similarly -> similar. Also, I’m not sure why this is summarized as “largely similar results” when the main effect of condition has disappeared. This could be stated as “results consistent with the observation that the E+H condition was stronger than E-only or H-only”.

We have corrected this typo, and updated the wording to clarify that the main effect of fixation-condition is no longer significant (lines 333-336).
