# Supplementary material for: Multiple spatial reference frames underpin perceptual recalibration to audio-visual discrepancies
Source: PLoS One. 2021 May 17;16(5):e0251827. doi: 10.1371/journal.pone.0251827 (PMC8128243; doi:10.1371/journal.pone.0251827)
Supplement: Attachment — Submitted filename: PONE-D-21-03641_Review.docx [file pone.0251827.s012.docx]

This study presents two experiments attempting to disentangle the contributions of eye-centric and head-centric reference frames to the VAE, and to determine whether the relative contributions of these reference frames changes with continued exposure to an AV offset. Experiment 1 contains the key manipulations attempting to break the consistency of the AV offset in each frame independently; Experiment 2 is a control study designed to test what influence such inconsistency in AV offset has on the strength of the VAE. Overall, I found the paper clear and well-written, the data a valuable contribution, and the statistics thorough and above-board. I have some suggestions regarding terminology and the interpretation of some key results, but I think the addition of the second experiment strengthens the study overall. [As an aside, I was not a reviewer for the initial submission of this manuscript.]

**Major issues**

1) “*Coherent* AV disparity” is not a very clear term.

It took until I was well into the paper to understand what you meant by it. All your stimuli are in a sense “spatially incoherent” – the perceptual system is recalibrating to something perceived to have gone wrong, after all. I should also mention that in my corner of the multisensory perception field, coherence is a borderline reserved term referring to *temporal* coherence (e.g. the co-modulation of contrast and amplitude you used to encourage AV binding). I would suggest that you replace this term with “consistent AV disparity” (or similar) whenever possible to promote general readability.

2a) Is the VAE really “reduced” or “weakened” in Exp. 1?

In the EC and HC conditions, in which the consistent AV offset was disrupted in one of the two reference frames, the VAE was reduced when the adaptation duration was relatively short. However, in both of these conditions, the VAE eventually recovered to the same magnitude as the E+H condition by the longest adaptation duration. You do mention this point (e.g. L541), but to me, the terms “reduced” or “weakened” used elsewhere don’t accurately capture the data. In the EC and HC conditions, it’s not really that the magnitude of the VAE was reduced, but that it took more exposure to reach that maximum magnitude. This is in contrast to the results of Exp. 2, which showed a truly reduced VAE across all adaptation durations tested. Being more explicit about this difference might actually work to the benefit of your main claims (see next point).

2b) Could Exp. 2 be framed as breaking the consistency of the AV offset in *both* reference frames?

You claim that Exp. 2 is set up to parallel the E+H condition of Exp. 1 (e.g. L379). Here (and also at L502), you say that “both eye-and head-centered reference frames [were] matched.” It’s not entirely clear what is meant by “matched” here, but I think it means that the variability in AV offset was the same whether the visual stimuli are considered in EC or HC coordinates. But put another way, this means that there was no consistent AV disparity in either reference frame (i.e., as I understand it, this was an Eye+Head **In**coherent condition). Unlike the EC and HC conditions of Exp. 1, now the VAE never recovered at any adaptation duration tested. If this description is valid, then what you have is a delayed VAE if consistency is disrupted in either the HC or EC reference frame (as if the system can recover from ambiguity in one frame), and a permanently reduced VAE if you disrupt the consistent AV offset in both frames (Exp. 2). In any case, a more explicit explanation of this result is important – why did the VAE eventually recover in the EC and HC conditions, but not in Exp. 2, despite your attempt to match the extent of variability in AV offset?

On the topic, I have a somewhat nitpicky issue with your framing of Exp. 2. As you know, the inconsistent frames in your EC and HC conditions include zero-disparity AV exposures, wrong-direction exposures, as well as exposures to AV offsets so large as to potentially fall outside the spatial window of visual influence over auditory perception. It would have been shocking if these factors did not reduce the VAE, but you needed to show conclusively that they do. All fine. But in your setup to Exp. 2 you write, for instance, “the precise magnitude of VAEs expected following adaptation to variable disparities remains unclear.” The implication is that you’ll be doing some careful manipulation to determine what types of variability affect the VAE, when in reality you’re smashing it with a hammer to make sure it breaks (as needed for compatibility with Exp. 1). I would appreciate this experiment being set up something more like:

- [Describe previous work investigating the effect of spatial variability (Bruns et al.)]
- However, in the EC and HC conditions of our study, variability in the inconsistent frames was much larger than what has been previously investigated, and included zero-crossings.
- We needed to measure whether this large extent of variability negatively impacts the VAE, as we assumed in our design.

3) Potential recalibration of auditory spatial perception in conditions with changing eye position could be more thoroughly addressed.

In a couple instances, you mention that auditory spatial receptive fields are known to shift with changes in eye position. Importantly, this also manifests in human behavior, with auditory spatial perception shifting by up to about 40% in the direction of eye gaze. The time constant of this process varies widely across participants, but averages on the order of a couple minutes (Razavi et al. J. Neuro, 2007).

Specifically, I’m wondering about this in the context of whether the visual stimulus locations were randomized within an adaptation cycle, or if they proceeded in a set order, e.g. left to right. [Unless I missed it, please clearly state which of these was the case in your methods.] If the visual stimulus was in the same hemifield for multiple consecutive stimuli, then although the drive to shift auditory space would change depending on stimulus eccentricity, it would be in the same direction and thus possibly have a compounding effect across stimulus presentations. This would systematically alter the perceived AV disparity, shrinking or magnifying it depending on hemifield and condition.

In reality, I think even if multiple consecutive adaptations occurred in the same hemifield, there wouldn’t be enough time for these eye position effects to drastically alter your results. Nonetheless, I would appreciate it if the possibility were addressed in the discussion.

4) I don’t think 10s between adaptation cycles should reset the VAE at all close to baseline.

In fact, during a relatively short period of auditory localization following exposure to an AV offset, the VAE doesn’t necessarily decline toward baseline at all:

Blue = pre-exposure localization, grey = localization during AV exposure, red = post-exposure localization. Red period is roughly a couple minutes of auditory localization. From Bosen et al., PLoS one, 2018. [Full disclosure: I am a coauthor on this study, but you already cited it anyway.]

Because of this, I would guess that you actually have compounding adaptation across the 4 cycles within each block. One way to check for this (and a possible supplemental figure) would be to separately analyze the data from each cycle, to see if your measured VAE strengthens from first to last. If adaptation is compounding across cycles, it would at least be happening across all four adaptation durations (e.g., the proportional difference between the shortest and longest adaptation durations remains the same). However, if there’s sufficient data, looking at just the first adaptation-test cycle within the shortest adaptation condition could be an informative way to test your reference frame effects at the shortest possible time point.

Maximal (possibly overkill) suggestion: Test whether adaptation appears to compound across cycles, and separately analyze data from just the first cycle.

Intermediate suggestion: Test and report whether adaptation compounds across cycles, discuss whether this has any influence on your conclusions.

Minimal request: Discuss the possibility and how it might have influenced your results.

One final note in this section: While eye tracking would have been ideal, I do not think it is as critical as the previous reviewer did. Its primary value would probably have been in ensuring that participants were looking at the correct stimulus or fixation point, more so than assessing the precise accuracy of their fixation. Given the wide spacing between stimulus locations and the ample time participants have to correct initial saccade errors prior to stimulus presentation, fixation should have been accurate enough as long as participants followed directions.

**Small things**

A possible discussion point: Some studies have shown the VAE to be limited to the region of space where AV exposure occurred (e.g. Bruns & Röder, Psychological Res., 2019; earlier work cited in the intro of that paper). I’m curious what this means in the context of exposures where stimuli in one modality are spatially variable, while stimuli in the other modality are fixed in physical space (as is the case in your EC condition of Exp. 1). Does this affect how much the VAE generalizes during the test phase? I could take this or leave it in your discussion, though; it’s not as if you’re short for content.

L113) Please report participant demographics for the final pool after rejections, instead of / in addition to the pre-rejection demographics.

L228) So participants moved the visual marker and registered responses using peripheral vision? Just checking this.

L428) Figures 2a and 2b referenced in the text should be Figures 3a and 3b.
