# Supplementary material for: Multiple spatial reference frames underpin perceptual recalibration to audio-visual discrepancies
Source: PLoS One. 2021 May 17;16(5):e0251827. doi: 10.1371/journal.pone.0251827 (PMC8128243; doi:10.1371/journal.pone.0251827)
Supplement: Attachment — Submitted filename: reviewer_response.docx [file pone.0251827.s013.docx]

Reviewer #1: This study presents two experiments attempting to disentangle the contributions of eye-centric and head-centric reference frames to the VAE, and to determine whether the relative contributions of these reference frames changes with continued exposure to an AV offset. Experiment 1 contains the key manipulations attempting to break the consistency of the AV offset in each frame independently; Experiment 2 is a control study designed to test what influence such inconsistency in AV offset has on the strength of the VAE. Overall, I found the paper clear and well-written, the data a valuable contribution, and the statistics thorough and above-board. I have some suggestions regarding terminology and the interpretation of some key results, but I think the addition of the second experiment strengthens the study overall. [As an aside, I was not a reviewer for the initial submission of this manuscript.]

Major issues

1) “Coherent AV disparity” is not a very clear term.

It took until I was well into the paper to understand what you meant by it. All your stimuli are in a sense “spatially incoherent” – the perceptual system is recalibrating to something perceived to have gone wrong, after all. I should also mention that in my corner of the multisensory perception field, coherence is a borderline reserved term referring to temporal coherence (e.g. the co-modulation of contrast and amplitude you used to encourage AV binding). I would suggest that you replace this term with “consistent AV disparity” (or similar) whenever possible to promote general readability.

We have updated the terminology throughout to use “consistent” instead of “coherent”. We have also tried to help clarify the term by making more explicit that it is referring to the fact that a consistent audio-visual spatial disparity is obtained when using visual signals from a given reference space (e.g. lines 97-101).

2a) Is the VAE really “reduced” or “weakened” in Exp. 1?

In the EC and HC conditions, in which the consistent AV offset was disrupted in one of the two reference frames, the VAE was reduced when the adaptation duration was relatively short. However, in both of these conditions, the VAE eventually recovered to the same magnitude as the E+H condition by the longest adaptation duration. You do mention this point (e.g. L541), but to me, the terms “reduced” or “weakened” used elsewhere don’t accurately capture the data. In the EC and HC conditions, it’s not really that the magnitude of the VAE was reduced, but that it took more exposure to reach that maximum magnitude. This is in contrast to the results of Exp. 2, which showed a truly reduced VAE across all adaptation durations tested. Being more explicit about this difference might actually work to the benefit of your main claims (see next point).

2b) Could Exp. 2 be framed as breaking the consistency of the AV offset in both reference frames?

You claim that Exp. 2 is set up to parallel the E+H condition of Exp. 1 (e.g. L379). Here (and also at L502), you say that “both eye-and head-centered reference frames [were] matched.” It’s not entirely clear what is meant by “matched” here, but I think it means that the variability in AV offset was the same whether the visual stimuli are considered in EC or HC coordinates. But put another way, this means that there was no consistent AV disparity in either reference frame (i.e., as I understand it, this was an Eye+Head Incoherent condition). Unlike the EC and HC conditions of Exp. 1, now the VAE never recovered at any adaptation duration tested. If this description is valid, then what you have is a delayed VAE if consistency is disrupted in either the HC or EC reference frame (as if the system can recover from ambiguity in one frame), and a permanently reduced VAE if you disrupt the consistent AV offset in both frames (Exp. 2). In any case, a more explicit explanation of this result is important – why did the VAE eventually recover in the EC and HC conditions, but not in Exp. 2, despite your attempt to match the extent of variability in AV offset?

These are good points. We have updated our General Discussion to note that the results of Experiment 1 are more consistent with a delay in the saturation point of the VAE, rather than necessarily a global reduction in the magnitude (lines 591-595). We have also noted that VAEs are more globally reduced in Experiment 2 (lines 637-643), though we have also caveated this to note that this could still be consistent with a simple delay in the saturation point but one which goes beyond our longest adaptation period (i.e. the VAE may eventually recover, but requires longer exposures than included here). We have also updated the terminology throughout to refer to the VAEs in Experiment 1 as being “disrupted”, rather than necessarily “reduced” or “weakened”.

By “matched” we meant that both eye- and head-centred visual signals in Experiment 2 will yield equivalent audio-visual disparities (but which vary over trials). We have updated the terminology throughout to clarify this (e.g. lines 410-412, but also elsewhere). The “eye+head-inconsistent” descriptor is also accurate, so we have included this in a few places too.

On the topic, I have a somewhat nitpicky issue with your framing of Exp. 2. As you know, the inconsistent frames in your EC and HC conditions include zero-disparity AV exposures, wrong-direction exposures, as well as exposures to AV offsets so large as to potentially fall outside the spatial window of visual influence over auditory perception. It would have been shocking if these factors did not reduce the VAE, but you needed to show conclusively that they do. All fine. But in your setup to Exp. 2 you write, for instance, “the precise magnitude of VAEs expected following adaptation to variable disparities remains unclear.” The implication is that you’ll be doing some careful manipulation to determine what types of variability affect the VAE, when in reality you’re smashing it with a hammer to make sure it breaks (as needed for compatibility with Exp. 1). I would appreciate this experiment being set up something more like:

• [Describe previous work investigating the effect of spatial variability (Bruns et al.)]

• However, in the EC and HC conditions of our study, variability in the inconsistent frames was much larger than what has been previously investigated, and included zero-crossings.

• We needed to measure whether this large extent of variability negatively impacts the VAE, as we assumed in our design.

These are fair points, and we had not intended to suggest the second experiment would investigate multiple levels of variability. We have updated the paragraph leading into Experiment 2 (lines 395-419) in line with your suggestions.

3) Potential recalibration of auditory spatial perception in conditions with changing eye position could be more thoroughly addressed.

In a couple instances, you mention that auditory spatial receptive fields are known to shift with changes in eye position. Importantly, this also manifests in human behavior, with auditory spatial perception shifting by up to about 40% in the direction of eye gaze. The time constant of this process varies widely across participants, but averages on the order of a couple minutes (Razavi et al. J. Neuro, 2007).

Specifically, I’m wondering about this in the context of whether the visual stimulus locations were randomized within an adaptation cycle, or if they proceeded in a set order, e.g. left to right. [Unless I missed it, please clearly state which of these was the case in your methods.] If the visual stimulus was in the same hemifield for multiple consecutive stimuli, then although the drive to shift auditory space would change depending on stimulus eccentricity, it would be in the same direction and thus possibly have a compounding effect across stimulus presentations. This would systematically alter the perceived AV disparity, shrinking or magnifying it depending on hemifield and condition.

In reality, I think even if multiple consecutive adaptations occurred in the same hemifield, there wouldn’t be enough time for these eye position effects to drastically alter your results. Nonetheless, I would appreciate it if the possibility were addressed in the discussion.

The order of the locations was randomised within each adaptation cycle – we have updated the methods to include this detail (lines 223-224; line 457). We have further included references to the Razavi *et al.* paper in our General Discussion (lines 664-665; 678-682) and noted that they demonstrate perceptual changes occurring at a much slower timescale than the speed of fixation changes within our paradigm.

4) I don’t think 10s between adaptation cycles should reset the VAE at all close to baseline.

In fact, during a relatively short period of auditory localization following exposure to an AV offset, the VAE doesn’t necessarily decline toward baseline at all:

Blue = pre-exposure localization, grey = localization during AV exposure, red = post-exposure localization. Red period is roughly a couple minutes of auditory localization. From Bosen et al., PLoS one, 2018. [Full disclosure: I am a coauthor on this study, but you already cited it anyway.]

Because of this, I would guess that you actually have compounding adaptation across the 4 cycles within each block. One way to check for this (and a possible supplemental figure) would be to separately analyze the data from each cycle, to see if your measured VAE strengthens from first to last. If adaptation is compounding across cycles, it would at least be happening across all four adaptation durations (e.g., the proportional difference between the shortest and longest adaptation durations remains the same). However, if there’s sufficient data, looking at just the first adaptation-test cycle within the shortest adaptation condition could be an informative way to test your reference frame effects at the shortest possible time point.

Maximal (possibly overkill) suggestion: Test whether adaptation appears to compound across cycles, and separately analyze data from just the first cycle.

Intermediate suggestion: Test and report whether adaptation compounds across cycles, discuss whether this has any influence on your conclusions.

Minimal request: Discuss the possibility and how it might have influenced your results.

The gaps between adaptation phases will in practice be somewhat longer than 10 seconds as they also include the time taken to complete the test phases. Nevertheless, we can see there is still the potential for adaptation effects to carry over between successive cycles, so we analysed this explicitly by re-running our first-level regression analyses and then estimating VAE magnitudes for each cycle separately. We have illustrated the results in a new supplementary figure (*S6 Fig*).

We also repeated our main ANOVAs with the cycle-number included as an additional repeated-measures factor. For Experiment 1, this showed no significant main effect of the cycle-number and the Bayes factor indicated substantial support for the null hypothesis (F(2.73, 51.81) = 2.10, p = .117, BF_10_ = 0.01), and nor did it show a significant interaction with any of the other factors (all *p* > .05, all BF_10_ < 0.33). Importantly, the principal results from our original analyses still held: there was a significant main effect of fixation-condition (F(1.62, 30.69) = 5.04, *p* = .018, BF_10_ = 1523.30), but no significant main effect of adaptation-duration (F(2.26, 42.93) = 2.11, *p* = .128, BF_10_ = 2.09) and no fixation-condition by adaptation-duration interaction (F(4.32, 82.08) = 140.27, *p* = .440, BF_10_ = 0.09). Similarly for Experiment 2, there was no significant main effect of the cycle-number (F(2.25, 67.36) = 1.76, *p* = .175, BF_10_ = 0.05) and no significant interactions with any of the other factors (all *p* > .05, all BF_10_ < 0.33). Again, the principal results of the original analyses still held: there was a significant main effect of disparity-type (F(1, 30) = 9.82, *p* = .004, BF_10_ = 9.73), but no significant main effect of adaptation-duration (F(2.48, 74.29) = 0.16, *p* = .896, BF_10_ = 0.01) and no significant disparity-type by adaptation-duration interaction (F(2.48, 74.29) = 0.18, *p* = 0.878, BF_10_ = 0.03).

Thus, the cycle number did not have a significant effect on the VAE magnitudes in either experiment, and our principal results hold when analysed across cycles. Whilst we cannot entirely discount the possibility of adaptation effects carrying over between cycles, it appears that if any such effects did occur then they did not substantially influence our results and would not alter our conclusions.

One final note in this section: While eye tracking would have been ideal, I do not think it is as critical as the previous reviewer did. Its primary value would probably have been in ensuring that participants were looking at the correct stimulus or fixation point, more so than assessing the precise accuracy of their fixation. Given the wide spacing between stimulus locations and the ample time participants have to correct initial saccade errors prior to stimulus presentation, fixation should have been accurate enough as long as participants followed directions.

Small things

A possible discussion point: Some studies have shown the VAE to be limited to the region of space where AV exposure occurred (e.g. Bruns & Röder, Psychological Res., 2019; earlier work cited in the intro of that paper). I’m curious what this means in the context of exposures where stimuli in one modality are spatially variable, while stimuli in the other modality are fixed in physical space (as is the case in your EC condition of Exp. 1). Does this affect how much the VAE generalizes during the test phase? I could take this or leave it in your discussion, though; it’s not as if you’re short for content.

The biases in localisation responses occur reasonably uniformly across space in all conditions. In the case of the eye-consistent condition, this would suggest a generalisation at least across the space covered by the adapting visual stimuli even though the adapting auditory stimulus was fixed in space. To illustrate this a little more clearly, we have updated the regression model plots (S1-S4 Figs) to also plot the model residuals over stimulus azimuths, and noted these in the General Discussion (lines 606-610). If response biases had been localised to specific regions of space this would result in a non-uniform distribution of residuals over azimuths. Instead, the residuals in each condition appear reasonably evenly distributed across azimuths, indicating recalibration effects were fairly uniform across space. Nevertheless, a more proper investigation of this question would require a different study design to our own, ideally including test trials at locations outside the range of the adapting locations to quantify the extent of generalisation.

L113) Please report participant demographics for the final pool after rejections, instead of / in addition to the pre-rejection demographics.

We have made this change.

L228) So participants moved the visual marker and registered responses using peripheral vision? Just checking this.

Yes, participants maintained central fixation throughout the test phases – responses will have been made in the periphery. We have updated the methods to clarify this (lines 244-245).

L428) Figures 2a and 2b referenced in the text should be Figures 3a and 3b.

We have made this change.

Reviewer #2: This paper aimed at investigating the contributions of eye- vs. head-centered reference frames for crossmodal spatial recalibration in the ventriloquism aftereffect (VAE) paradigm. To this end, three adaptation conditions were compared, one in which EC and HC reference frames were aligned, one in which the eyes (but not the head) followed the visual component but the actual AV spatial disparity was preserved, and one in which the auditory stimulus was always presented at ±20° relative to the head regardless of the (fixated) visual stimulus location. It was found that the VAE was reduced but still significant in the latter two conditions, and this effect did not interact with adaptation duration. A control experiment found a reduced but significant VAE also in a condition in which EC and HC were aligned but actual AV spatial disparities differed as in the incoherent reference frames of the main experiment.

The paper has already been through one round of revisions and while reading the manuscript I had the same thought as the previous reviewer: Why would auditory information stay in an exclusively head-centered reference frame and why/how would head-centered auditory information be integrated with eye-centered visual information without prior remapping? I think it should be made clearer early on (i.e. explicitly mentioned already in the introduction and methods) that the experiment design is based on the assumption that auditory information stays exclusively HC but can nevertheless be combined with EC visual information and that any conclusions about contributions of reference frames are only valid if this assumption is true. That way, the readers could better judge for themselves what to make of the results.

Yes, our paradigm does critically assume that auditory signals remain in head-centred co-ordinates, while visual signals may be represented in either eye- or head-centred frames. We have updated the Introduction (lines 58-59, 96-100) and Experiment 1 Methods (lines 185-187) sections to clarify this. In our General Discussion, we already provided some consideration to the possibility of eye-centred transformations of auditory signals, and why we felt this was unlikely to account for our results (paragraph from lines 671-692); this section has now also been slightly expanded in response to comments by Reviewer 1.

Having said that, however, the discussion of whether HC auditory coordinates could be directly integrated with EC visual coordinates somewhat reminded me of the literature on tactile remapping with hand crossing manipulations where anatomical and external coordinates are brought into conflict (e.g., Yamamoto & Kitazawa, 2001, Nat Neurosci). Some studies have suggested that visual-external coordinates may be integrated with anatomical (i.e., non-remapped) tactile coordinates if processing time is short (e.g., Azanon & Soto-Faraco, 2008, Curr Biol) or if the presumed tactile remapping process is disrupted via TMS (Bolognini & Maravita, 2007, Curr Biol; Renzi et al., 2013, J Cogn Neurosci). Thus, I think that these findings do support the authors’ line of argument, but I would leave it up to them whether they want to discuss these findings giving that the present study did not involve any tactile stimuli.

We have added some references to these studies in our General Discussion (lines 668-670).

Moreover, I think the new control experiment at least partially addresses the issue and (in light of the Bruns et al. 2020 findings) the results of this new experiment are highly interesting in their own right. However, a statistical comparison of the new control condition is only carried out with the baseline condition in which both reference frames were aligned and I was wondering whether a statistical comparison with the other two conditions wouldn’t be informative as well? For example, one could have had the hypothesis that spatially incoherent in both RF leads to a stronger reduction of the VAE than incoherence in only one RF (which does not seem to be the case from visual inspection of the data).

VAEs overlaid from all conditions in both experiments are shown below. The VAEs elicited in Experiment 2 (when disparities are inconsistent under both reference spaces) are generally reduced in magnitude compared to all conditions from Experiment 1, however the differences from the eye- and head-consistent conditions are smaller than from the eye+head-consistent condition. Statistical comparisons using mixed-design ANOVAs revealed only marginal effects of disparity-type, and the corresponding Bayes factors appeared inconclusive. In detail, for the comparison of Experiment 2 against the eye-consistent Experiment 1 condition, there are no significant main effects of disparity-type (F(1, 30) = 3.50, *p* = .071, BF_10_ = 0.79) or adaptation-duration (F(2.56, 76.74) = 0.82, *p* = .468, BF_10_ = 0.20) and no significant interaction (F(2.56, 76.74) = 0.69, *p* = .539, BF_10_ = 0.29). Similarly, for the comparison of Experiment 2 against the head-consistent Experiment 1 condition, there are no significant main effects of disparity-type (F(1, 30) = 3.36, *p* = .079, BF_10_ = 0.82) or adaptation-duration (F(2.45, 73.53) = 0.71, *p* = .521, BF_10_ = 0.18) and no significant interaction (F(2.45, 73.53) = 0.84, *p* = .456, BF_10_ = 0.34).

Our reason for omitting these comparisons from the manuscript is that they are not entirely fair because Experiment 2 is only designed to match the eye+head-consistent condition from Experiment 1. The results of Experiment 2 demonstrate that the effects observed in Experiment 1 cannot be accounted for purely by task differences (e.g. differences in fixation behaviours), but we do not claim that such differences couldn’t have had any effect. Equally well then, if we had included further conditions within the second experiment which matched the requirements of the eye- and head-consistent conditions (e.g. movement of the fixation position) this might have reduced the VAE magnitudes even further. However, as we did not include such conditions, we have restricted the reported results to only the comparison with the eye+head-consistent condition that Experiment 2 was designed to match.

There may be other implications of the results worthy being pointed out in the discussion. For example, many studies of the VAE did not control for eye movements during the exposure phase. The results of the head-coherent condition may suggest that these studies may nevertheless provide valid estimates of the VAE.

This is an interesting point. We have added a sentence to our General Discussion (lines 595-598) pointing this out.

Another thought I had was whether the design with only very short (1 min) breaks between different adaptation duration blocks is really suitable to investigate effects of adaptation duration. Newer findings suggest that there will likely be carry-over effects between blocks (e.g., Bruns & Röder, 2019, JEP:HPP) and I wonder to what extent such carry-over effects might have obscured any effect of adaptation duration within a particular block?

The gaps between blocks were enforced as a minimum of 1 minute, but participants could (and frequently did) take longer. Furthermore, participants could (and did) complete the experiment across multiple sessions, in which case there could be delays of several hours or even days between some blocks. Finally, the block order (and hence also condition order) was randomised for each participant, thus any carryover adaptation effects between blocks would not have influenced the results in any consistent manner across conditions, and hence would average out over participants. While we cannot entirely discount the possibility of carryover adaptation effects between blocks, we feel it is unlikely that such effects could explain our current pattern of results or would affect our conclusions.

Other than the points mentioned, I think this is a really nice and technically sound paper which is very well-written and uses well-justified statistical analyses, and I have no further comments of substance.

Thank you.
